# Therapeutic Potential of Stem Cell-Derived Extracellular Vesicles in Liver Injury

**DOI:** 10.3390/biomedicines12112489

**Published:** 2024-10-30

**Authors:** Jingjing Dong, Ying Luo, Yingtang Gao

**Affiliations:** 1 School of Medicine, Nankai University, Tianjin 300071, China; dongjingjing2022@mail.nankai.edu.cn; 2 Tianjin Key Laboratory of Extracorporeal Life Support for Critical Diseases, Tianjin Institute of Hepatobiliary Disease, Nankai University Affiliated Third Center Hospital, Tianjin 300170, China; ly200101042@hotmail.com

**Keywords:** extracellular vesicles, stem cells, liver injury, animal models, clinical research

## Abstract

Liver injury caused by various factors significantly impacts human health. Stem cell transplantation has potential for enhancing liver functionality, but safety concerns such as immune rejection, tumorigenesis, and the formation of emboli in the lungs remain. Recent studies have shown that stem cells primarily exert their effects through the secretion of extracellular vesicles (EVs). EVs have been shown to play crucial roles in reducing inflammation, preventing cell death, and promoting liver cell proliferation. Additionally, they can function as carriers to deliver targeted drugs to the liver, thereby exerting specific physiological effects. EVs possess several advantages, including structural stability, low immunogenicity, minimal tumorigenicity targeting capabilities, and convenient collection. Consequently, EVs have garnered significant attention from researchers and are expected to become alternative therapeutic agents to stem cell therapy. This article provides a comprehensive review of the current research progress in the use of stem cell-derived EVs in the treatment of liver injury.

## 1. Introduction

Liver injury can arise from various causes, such as drug toxicity, surgical resection, oxidative stress, and inflammatory reactions after liver transplantation. These injuries often result in severe liver dysfunction, thereby exerting significant effects on the quality of life experienced by affected patients [1,2].

The use of stem cells, which are characterized by their pluripotent differentiation potential and self-renewal ability, has emerged as a promising approach in the field of liver disease therapy. Extensive animal experiments and clinical studies have demonstrated the therapeutic efficacy of stem cell transplantation in conditions such as liver ischemia–reperfusion injury (IRI) [3], liver fibrosis [4,5], and liver cancer [6]. Commonly used stem cell types include embryonic stem cells, hematopoietic stem cells, mesenchymal stem cells, liver stem cells, and induced pluripotent stem cells [7]. The mechanisms underlying the therapeutic effects of stem cell therapy include the induction of endogenous cell proliferation, the inhibition of cell apoptosis, and immune regulation [8]. However, several challenges need to be addressed in stem cell therapy, such as the limited in vivo survival time of transplanted stem cells [9], the low homing efficiency [10], immune rejection [11], and the risk of pulmonary embolism following intravenous injection [12,13].

In recent years, research has highlighted the crucial role of extracellular vesicles (EVs) secreted by stem cells in mediating therapeutic effects rather than relying solely on the differentiation of stem cells into functional cells [14,15,16,17,18]. Stem cell-derived EVs (SC-EVs) retain similar contents as their parent cells, endowing them with biological functions akin to those of the original stem cells [19,20,21]. Compared to stem cells, SC-EVs offer several advantages: (1) a smaller size, thereby preventing entrapment and thrombus formation within the microvasculature [22]; (2) precise localization to the liver after intravenous injection [23]; (3) flexible dosage adjustments [23]; (4) lower immunogenicity due to decreased levels of membrane-bound proteins [24]; (5) minimal tumorigenicity due to the absence of cellular components [25]; and (6) a relatively simple structure that allows for modifications to confer specific biological functions for precise therapies [26]. Consequently, the use of SC-EVs has emerged as a promising alternative therapeutic strategy to conventional stem cell therapy.

**Figure 1:**
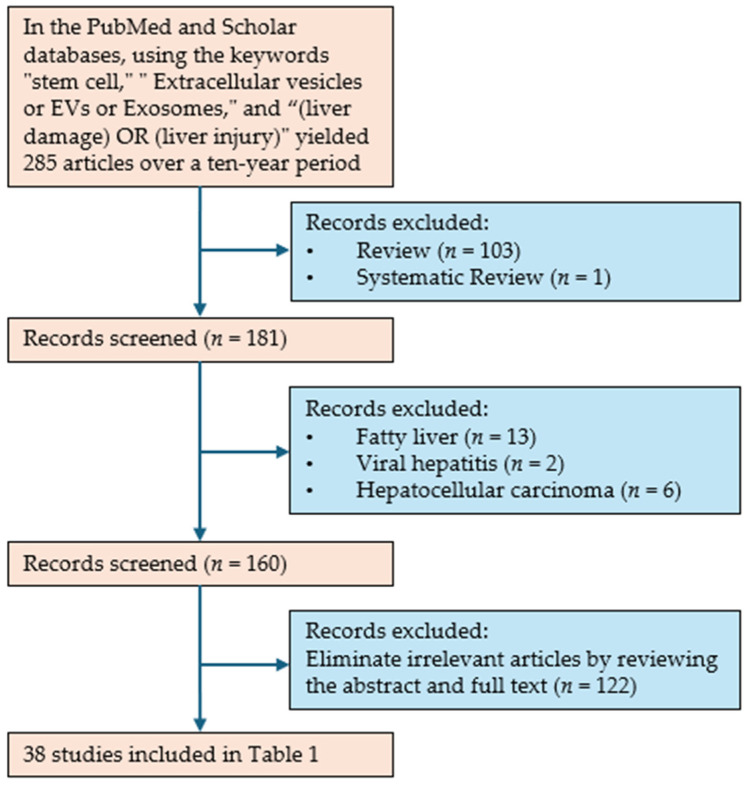
Flowchart of study selection for Table 1.

In the PubMed and Scholar databases, a search was conducted using the keywords “stem cell”, “Extracellular vesicles or EVs or Exosomes”, and “(liver damage) OR (liver injury)”, yielding 285 articles over a ten-year period. The specific screening process is shown in Figure 1. At least two authors collaborated to select 38 original research articles, which are summarized in Table 1. This review provides a comprehensive summary of the current research progress in the application of SC-EVs in the treatment of liver injury.

## 2. Extracellular Vesicles

EVs are membrane-bound vesicles released by various cell types that contain a diverse array of biomolecules, such as nucleic acids (DNA, mRNA, lncRNA, microRNA), proteins, peptides, lipids, and other bioactive molecules [60,61]. EVs play crucial roles in intercellular communication, cell growth, apoptosis inhibition, angiogenesis, and immune regulation by transferring their cargo to target cells through fusion with the target cell membrane, endocytosis, or binding to surface receptors on target cells [62]. Based on their biogenesis mechanisms, EVs can be classified into three main types: exosomes, microvesicles, and apoptotic bodies [63]. Exosomes are formed through the invagination of the plasma membrane, leading to the creation of early endosomes that mature into late endosomes, eventually developing into multivesicular bodies (MVBs). These MVBs then fuse with the plasma membrane, releasing exosomes with diameters of approximately 30–150 nm [64,65]. Microvesicles, ranging from 50 to 1000 nm in diameter, are directly budded and released from the plasma membrane of live cells. Apoptotic bodies, which are the largest type of EV with diameters of approximately 800–5000 nm, originate from apoptotic cells [66].

While most studies classify vesicles containing exosome-like proteins as exosomes [40,55,56], there is currently no standardized method to fully distinguish exosomes, microvesicles, and apoptotic bodies [67]. Moreover, other types of extracellular vesicles may exist beyond the known classifications [68]. Therefore, to avoid controversy, this article collectively refers to exosomes and other extracellular vesicles as EVs.

## 3. Therapeutic Studies of SC-EVs In Vitro and in Animal Models of Liver Injury

**Figure 2:**
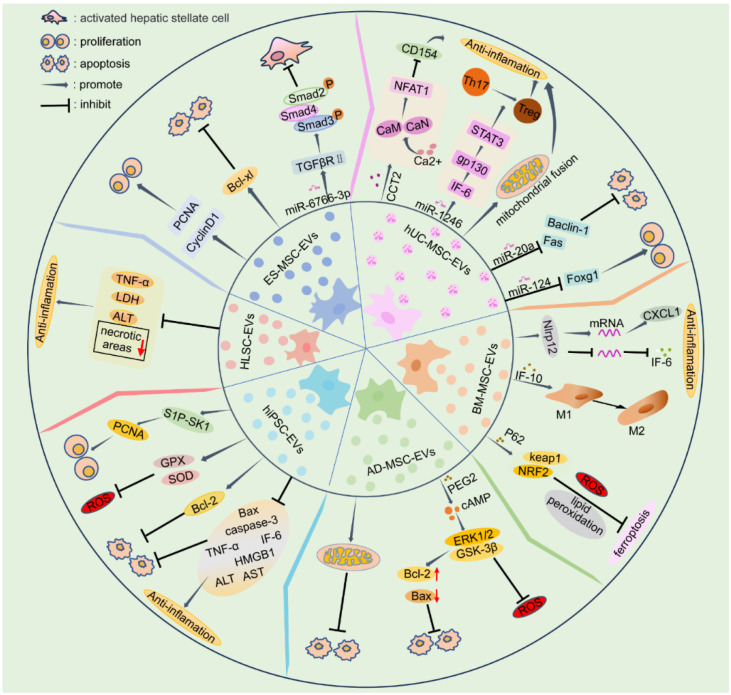
The reparative mechanisms of six types of stem cell-derived extracellular vesicles (SC-EVs) targeting liver injury. 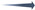: promote; 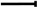: inhibit.

Liver damage caused by ischemia–reperfusion injury (IRI) is a significant consequence of liver resection and transplantation. During hepatic ischemia, hepatocytes experience hypoxia, which leads to cell death. When the extent of hepatocyte death surpasses the regenerative capacity of the liver, severe impairment of liver function occurs [69]. In response to reperfusion, reactive oxygen species (ROS) and inflammatory factors are produced, promoting hepatocyte necrosis and apoptosis and thereby aggravating liver injury [70]. In recent years, numerous studies have provided preclinical evidence supporting the efficacy of SC-EVs in treating liver injury. Six types of SC-EVs have been shown to have therapeutic effects on liver injury in vitro and in rodent models (Figure 2 and Table 1). Different tissue-derived SCs and their secreted EVs exhibit similar significant therapeutic effects in alleviating liver injury. Moreover, the therapeutic efficacy of SC-EVs can be enhanced through specific preconditioning methods [28,38,42]. This article primarily focuses on the use of six specific types of SC-EVs to treat liver injury.

### 3.1. EVs Derived from Embryonic Stem Cell-Derived Mesenchymal Stem Cells (ES-MSCs)

Tan et al. [2] demonstrated the protective effect of EVs derived from human embryonic stem cell-derived mesenchymal stem cells (hES-MSCs) in acute liver injury induced by carbon tetrachloride (CCl4) in mice, which is a classic liver toxicity model. After intrasplenic injection of EVs, the levels of proliferating cell nuclear antigen (PCNA) and cyclin D1 significantly increased in the liver, and the expression of the antiapoptotic protein Bcl-xl increased. These results suggest that EVs derived from hES-MSCs can promote hepatocyte proliferation and inhibit apoptosis to restore liver regeneration. However, no significant effect on oxidative stress was observed when liver injury was treated with EVs derived from hES-MSCs, which may be related to the rapid degradation and significant reduction in GPX1 in embryonic stem cells during early differentiation [71]. Moreover, cells cultured in a 3D environment exhibit stronger regenerative abilities than those cultured in a 2D environment [27]. Moreover, do EVs derived from 3D culture models possess greater potential? Wang et al. [27] conducted relevant studies and confirmed that EVs derived from human embryonic stem cells cultured in a 3D model (3D-hESC-EVs) accumulated more efficiently in the liver and exhibited more significant therapeutic potential in a mouse model of liver injury than those cultured in 2D conditions. The main mechanism may involve the transfer of miR-6766-3p, which is abundant in 3D-cultured hESC-EVs, to activated hepatic stellate cells (HSCs). This transfer inhibits the expression of *TGFβRII* and downstream SMAD proteins, including the phosphorylated proteins p-SMAD2/3 and SMAD4, preventing their oligomerization. Consequently, HSC activation is reduced, inhibiting the progression of liver fibrosis.

Compared to EVs secreted by bone marrow- and adipose-derived MSCs, EVs derived from ES-MSCs have a more robust ability to secrete anti-inflammatory cytokines and inhibit the proliferation of peripheral blood mononuclear cells (PBMCs) [19]. Therefore, in terms of regulating immune cell activity and reducing inflammation, EVs derived from ES-MSCs may be a more advantageous choice.

### 3.2. EVs Derived from Human Umbilical Cord Mesenchymal Stem Cells (hUC-MSCs)

EVs derived from hUC-MSCs possess protective effects against liver injury induced by ischemia–reperfusion [28]. CD4+ T cells are critical for the initiation of the inflammatory response in liver IRI. Protein mass spectrometry analysis showed that EVs derived from hUC-MSCs were rich in TCP1 subunit 2 (CCT2). CCT2 in EVs acted on CD4+ T cells by targeting the Ca^2+^-calmodulin-NFAT1 signaling pathway, inhibiting the expression of inflammatory factors and CD154, which initiates liver inflammation; thus, the initiation of inflammation was blocked [29]. The balance between proinflammatory Th17 cells and anti-inflammatory Treg cells is a key factor in the development of liver IRI [72]. Xie et al. [30] demonstrated that miR-1246 in hUC-MSC-derived EVs could promote the transformation of Th17 cells to Treg cells by mediating the IL-6-gp130-STAT3 axis in CD4+ T cells, thereby reducing inflammation and improving liver IRI. Consistent with these animal experimental results, EVs also significantly reduced the levels of proinflammatory cytokines (TNF-α, IL-6a, and IL-1β) in a cell model of hypoxia/reoxygenation (H/R) by delivering miR-1246, thereby alleviating inflammation [15].

Autophagy and apoptosis are also important pathological mechanisms of liver IRI. *Fas* is thought to induce liver cell apoptosis [73], and *Beclin-1* is a key factor in autophagy [74]. In a liver IRI model, miR-20a expression was inhibited, and hUC-MSC-derived EVs contained high levels of miR-20a. It was found that hUC-MSC-derived EVs could inhibit *Fas* and *Beclin-1* expression by delivering miR-20a to target the 3′UTR, significantly reducing liver cell apoptosis and alleviating liver injury [31]. In addition, miRNAs in hUC-MSC-derived EVs can promote liver cell proliferation after partial hepatectomy. Song et al. [32] confirmed that miR-124 in EVs targeted the transcription factor *Foxg1* in liver cells and inhibited its expression, thereby promoting liver regeneration. In contrast, EVs with a miR-124 deficiency exhibited a weakened ability to promote cell regeneration. hUC-MSC-EVs contain abundant GPX1 and can exert their effects on liver cells by secreting GPX1 and inducing ERK1/2 phosphorylation, thereby alleviating liver oxidative damage and reducing cell apoptosis [35]. Subsequently, Jiang et al. once again verified the antioxidant capacity and antiapoptosis ability of EVs in an identical experimental model [75].

In the early stage of liver IRI, neutrophil infiltration in the liver increases, leading to an increase in neutrophil extracellular traps (NETs) in the liver [76]. It has been proven that hUC-MSC-derived EVs can reduce neutrophil infiltration, thereby reducing the inflammatory response [33]. Lu et al. [34] proposed that hUC-MSC-EVs exert protective effects on neutrophils in the liver and further explored the underlying mechanism, suggesting that hUC-MSC-EVs could induce mitochondrial fusion in neutrophils by transferring functional mitochondria, thus restoring mitochondrial function and reducing the formation of local NETs, playing a therapeutic role.

In summary, hUC-MSC-derived EVs can be used to treat liver injury by preventing inflammation, reducing autophagy and apoptosis, and promoting liver cell regeneration.

### 3.3. EVs Derived from Bone Marrow Mesenchymal Stem Cells (BM-MSCs)

EVs derived from BM-MSCs mainly achieve therapeutic effects and treat liver injury by alleviating inflammation and diminishing cell apoptosis, and different mechanisms of action have been observed in various liver injury models. In a mouse liver ischemia–reperfusion injury (IRI) model [36], EVs derived from mouse BM-MSCs were shown to exert anti-inflammatory effects primarily by targeting NACHT, LRR, and PYD domain-containing protein 12 (Nlrp12). Nlrp12 is a negative regulator of inflammatory activity in the immune system. After EVs targeted Nlrp12, the mRNA expression of Nlrp12 and chemokine (C-X-C motif) ligand 1 (CXCL1) increased, while the mRNA expression of several inflammatory factors, such as IL-6, was reduced during liver injury. This regulation of inflammatory factors occurs at the transcriptional level rather than by directly modulating their protein levels [37]. Zhang et al. [38] have demonstrated that BM-MSC-derived EVs ameliorate the degree of liver inflammation by inducing the upregulation of hepatocyte *FGF21* expression, which inhibits the *JAK2/STAT3* pathway. Moreover, the hepatoprotective effect of the EVs secreted by BM-MSCs can be improved by pretreating BM-MSCs with baicalin. Additionally, miRNA sequencing of BM-MSC-derived EVs has revealed an enrichment of miR-25-3p. Through a mouse model of hepatic ischemia–reperfusion injury (HIRI) and hypoxia/reoxygenation (H/R) cell models, it has been determined that miR-25-3p reduces hepatocyte apoptosis by downregulating the target gene *PTEN*, inhibiting the p53 signaling pathway, and promoting cell proliferation in vitro models [39]. In addition to hepatocytes, BM-MSC-derived EVs can also target Kupffer cells [77]. Zhang et al. [41] confirmed that mouse BM-MSC-derived EVs interacted with liver macrophages and promoted their anti-inflammatory polarization by delivering endogenous IL-10, thereby reducing liver inflammation.

In recent years, ferroptosis has been associated with acute liver injury (ALI) [78]. In a mouse model of ALI induced by D-galactosamine and lipopolysaccharide (D-GaIN/LPS), BM-MSC-derived EVs could inhibit ROS and lipid peroxidation-induced ferroptosis by activating the P62 protein-mediated Keap1-NRF2 pathway [42]. Furthermore, pretreatment of BM-MSC-derived EVs with baicalin increased the protein levels of P62 within EVs, resulting in increased inhibition of cell death and anti-inflammatory effects in vivo. Similarly, hBM-MSC-derived EVs pretreated with glycyrrhizic acid could more robustly regulate abnormal protein levels in vivo than hBM-MSC-derived EVs alone [43]. The level of the antiapoptotic protein Bcl-2 significantly increased, while the levels of inflammatory factors such as IL-1β and TNF-α decreased significantly. Additionally, Tamura et al. simulated the continuous release of EVs in vivo by administering multiple doses of mBM-MSC-derived EVs [23]. The authors demonstrated that multiple administrations of mBM-MSC-derived EVs were more effective than single administrations of either mBM-MSC-derived EVs or mBM-MSCs alone. Multiple administrations resulted in smaller liver necrotic areas, reduced levels of the liver injury marker ALT, and increased numbers of anti-inflammatory Treg cells, indicating improved therapeutic outcomes.

Furthermore, Yang et al. [40] induced the differentiation of mBM-MSCs into hepatocytes and extracted EVs from them. After the EVs were intravenously injected into mice, the levels of autophagy activity markers, such as LC3-II and Beclin-1, which are components of the PI3K complex required for autophagy, were increased, confirming an increase in cellular autophagy. Moreover, concentrated EVs were obtained through ultracentrifugation of EVS-rich MSC-CMs and then injected into a rat hepatic IRI model through the hepatic portal vein. Concentrated EVs showed stronger antioxidative stress and antiapoptotic cell ability [44]. These findings indicate an improvement in the ability of liver cells to remove damaged mitochondria, leading to reduced ROS production, the inhibition of liver cell apoptosis, and the alleviation of liver injury.

### 3.4. EVs Derived from Adipose-Derived Mesenchymal Stem Cells (AD-MSCs)

In a rat liver IRI model, the therapeutic effect of EVs secreted by AD-MSCs was similar to that of the two types of stem cell-derived EVs mentioned above. The mechanism may be related to the prostaglandin E2 (PGE2) protein contained in rAD-MSC-EVs. After PGE2 acts on target cells, it activates the second messenger cyclic adenosine monophosphate (cAMP), which in turn promotes the phosphorylation of extracellular regulated protein kinase (ERK) ERK1/2 and glycogen synthase kinase (GSK) GSK-3β, inhibits the production of ROS, and simultaneously increases the level of the antiapoptotic protein Bcl-2 and decreases the level of the proapoptotic protein Bax, thereby reducing oxidative stress and cell apoptosis [46]. Cellular homeostasis after ischemia–reperfusion is closely related to mitochondrial homeostasis [79]. rAD-MSC-derived EVs have been shown to promote mitochondrial fusion, inhibit mitochondrial fission, and enhance mitochondrial biogenesis, thereby regulating mitochondrial homeostasis and inhibiting cell apoptosis, which is beneficial for alleviating liver IRI [1].

In addition, mAD-MSC-EVs alleviate acute liver injury induced by CCL4, as indicated by decreases in the liver injury markers ALT, AST, and γ-GT and an increase in serum ALB levels [48]. Subsequently, the authors pretreated mAD-MSC-EVs with quercetin and vitamin A, and the reduction in liver injury markers was even more significant than that in control cells, indicating that pretreatment with EVs could increase their therapeutic efficacy and lead to new ideas for clinical disease treatment. Moreover, hAD-MSC-EVs could also promote liver cell proliferation, thus restoring liver function [47,49]. Additionally, in large animal models, AD-MSC-EVs have also demonstrated similar therapeutic effects on liver regeneration [50,51]. In a mini-pig model of hepatectomy combined with IRI, mini-pig AD-MSC-EVs, when administered intravenously, can target liver cells and ameliorate liver damage by inhibiting oxidative stress, promoting antiapoptotic effects, and alleviating endoplasmic reticulum stress reactions [45,52]. They also reduce the secretion of inflammatory factors and foster liver regeneration, thereby mitigating hepatic IRI [53]. Interestingly, a substantial body of experiments has confirmed that AD-MSC-EVs possess protective effects in vivo and in vitro that are similar to those of AD-MSCs [51,52,54]. These findings indicate that EVs hold significant potential as a novel cell-free therapeutic strategy and provide an experimental basis in animal models for the use of AD-MSC-EVs as an alternative to AD-MSCs for the treatment of hepatic IRI.

### 3.5. EVs Derived from Human Induced Pluripotent Stem Cell-Derived Mesenchymal Stromal Cells (hiPSC-MSCs)

In a liver IRI model, Nong et al. [55] showed that EVs secreted by hiPSC-MSCs could alleviate liver damage caused by liver IRI, which was mainly characterized by significant inhibition of liver injury markers (ALT, AST), apoptotic markers (caspase-3, bax), and inflammatory factors (TNF-α, IL-6, and HMGB1), while the levels of the antiapoptotic protein Bcl-2 and the antioxidant markers glutathione peroxidase (GPX) and superoxide dismutase (SOD) were significantly increased. These results indicate that EVs secreted by hiPSC-MSCs can alleviate liver damage by inhibiting liver cell apoptosis, reducing the inflammatory response after liver injury, and relieving oxidative stress. In addition, in an in vivo experiment, it was found that EVs secreted by hiPSC-MSCs could promote liver cell proliferation, possibly through activation of the sphingosine kinase and sphingosine-1-phosphate pathways by EVs in the liver, thereby promoting the expression of proliferating cell nuclear antigen (PCNA) and liver cell regeneration [56].

### 3.6. EVs Derived from Human Liver Stem Cells (HLSCs)

EVs derived from HLSCs can alleviate liver damage and promote liver cell proliferation. The optimal method for preserving transplanted tissue before liver transplantation is static cold storage (SCS). However, this method is not effective for preserving suboptimal transplants (such as livers donated after circulatory death); moreover, normothermic machine perfusion (NMP) can maintain the transplant at 37 °C, provide nutrients and oxygen, and has been proven to be an effective alternative method for preserving transplants [80,81]. Giving HLSC-EVs during the first 15 min of NMP can effectively reduce the release of the liver injury marker ALT and promote liver cell proliferation in a dose-dependent manner [57,58]. In contrast, in a mouse liver IRI model [59], after intravenous injection of HLSC-EVs, immunofluorescence analysis confirmed that the labeled HLSC-EVs were internalized by liver cells, leading to significant decreases in liver enzymes (such as ALT and LDH), the necrotic area, and certain cytokines (such as TNF-α). After the administration of higher doses of EVs, the changes in cytokine levels were more significant, indicating that the protective effect of HLSC-EVs against liver damage may be dose-dependent.

## 4. Clinical Research Progress on SC-EVs in Liver Diseases

A search for “liver diseases and stem cell” on the U.S. Clinical Trials website (clinicaltrials.gov) returned a total of 268 registered trials as of 6 June 2024. Then, out of 95 completed projects, 34 clinical trials of stem cell therapy for advanced liver disease, such as cirrhosis, were screened by browsing titles and abstracts. The most commonly used stem cell source is BM-MSCs, accounting for 35%, followed by hUC-MSCs and hematopoietic stem cells (HSCs), each accounting for 15%. Unfortunately, only two trials with published results, NCT04243681 and NCT00147043, have shown that the use of stem cell transplantation could be a secure and effective approach for individuals suffering from severe liver disorders [82,83]. However, the current clinical samples are too limited to demonstrate that this treatment method is sufficiently safe. For instance, stem cell transplantation may lead to a life-threatening complication, hepatic venous occlusive disease (VOD) [84].

SC-EVs are increasingly recognized for their superior safety profile. Unlike their parent cells, they cannot proliferate or trigger microvascular blockages, and they can be conveniently preserved without any degradation in their efficacy. On the U.S. Clinical Trials website, a search reveals a limited number of registered clinical trials utilizing SC-EVs for the treatment of liver disease, with only three trials currently documented in the database (Table 2). While two of these trials were registered by Sun Yat-sen University, they were later withdrawn due to challenges with EV supplies. The remaining clinical trial, which was registered in Iran, is still ongoing and has not yet published any results. In contrast, a search for “extracellular vesicles” and “exosomes” in the Chinese Clinical Trial Registry (chictr.org.cn) yielded 222 registered trials, 22 of which involved SC-EVs. There are three studies specifically focused on liver diseases (Table 2). Two of these trials aimed to evaluate the efficacy and safety of EVs for treating liver diseases, while the other focused on using tumor SC-EVs as drug carriers. In summary, clinical investigations into the realm of SC-EVs are in their nascent stages. Future multicenter, large-scale, randomized controlled trials will be critical to determine the safety and efficacy of SC-EVs.

## 5. Future Challenges in SC-EV Treatment of Liver Injury

Although SC-EVs have demonstrated potential in treating liver injury in animal trials, several challenges must be addressed for this cell-free therapy to emerge as an alternative treatment for liver diseases.

Firstly, to bolster the credibility of SC-EV treatments, a broad spectrum of cellular and preclinical trials is essential to elucidate the mechanisms of action. Secondly, there is a need to standardize and expand the production of EVs, as well as to ascertain the most efficacious administration pathways and dosages for various EV types. Thirdly, the current clinical research on the application of SC-EVs in hepatic disease therapy is rather constrained, with ongoing clinical trials characterized by limited participant numbers and a predominance of single-center studies. It is imperative that these hurdles be surmounted through additional clinical trials and scientific investigations, thereby enabling SC-EVs to evolve into a secure and potent treatment for liver damage.

## 6. Conclusions

SC-EVs have demonstrated potential as a treatment for liver injury, with supportive evidence from animal studies. Mesenchymal stem cell-derived EVs, including ES-MSCs, hUC-MSCs, BM-MSCs, AD-MSCs, and hiPSC-MSCs, as well as liver stem cell-derived EVs, have shown therapeutic benefits by reducing inflammation, improving liver cell viability, promoting regeneration and antioxidative stress, and reducing apoptosis. Despite promising preclinical findings, clinical research on SC-EVs for liver disease is limited, with trials featuring small samples and single-center studies. To enhance the clinical applicability of SC-EV therapy, further mechanistic studies, production standardization, and optimization of administration protocols are essential. In conclusion, SC-EVs may represent a promising, safer alternative to stem cell transplantation for liver injury treatment, pending further research and validation.

## Figures and Tables

**Table 1:** Experimental studies on the effects of EVs from different sources of stem cells on liver injury.

EV Source	Dose	Dose Frequency	Mode of Administration	Animal Model	In Vitro Experiments	Therapeutic Effect	Signaling Pathway/Mechanism	Reference
ES-MSC	0.4 μg/dose	single	Splenic injection	CCl_4_-induced liver injury mouse model	Yes	Increase hepatocyte proliferation (PCNA elevation); inhibit hepatocyte apoptosis	Not assessed	Tan et al., 2014 [2]
3D-hESC	100 μg/dose	twice a week, 4 weeks	Vein	CCl_4_-induced liver injury and fibrosis mouse model	Yes	3D-Exo has a better anti-inflammatory effect than 2D-Exoreduce HSC activation	miR-6766-3p inactivates smads signaling by restraining the expression of *TGFβRII*	Wang et al., 2021 [27]
ES-MSC	350 µg/dose	single	Splenic injection	Thioacetamide (TAA)-induced chronic liver injury rat model	Yes	Reduce inflammation; reduce apoptosis	Not assessed	Mardpour et al., 2018 [19]
hUC-MSC	100 μg/dose	single	Caudal vein	Liver IRI mouse model	Yes	Reduce inflammation; reduce apoptosis; inhibit oxidative stress	Inhibiting the NF-κB signaling pathway	Sameri et al., 2022 [28]
hUC-MSC	100 μg/dose	single	Caudal vein	Liver IRI mouse model	Yes	Inhibit the initiation of inflammatory responses	The Ca^2+^-calcineurin-NFAT1 signaling pathway	Zheng et al., 2020 [29]
hUC-MSC	10 μg/dose	single	Portal vein	Liver IRI mouse model	Yes	Reduce inflammation (decreased Th17/Treg ratio among CD4+ T cells)	Transfer miR-1246 targeting the IL-6-gp130-STAT3 pathway	Xie et al., 2019 [30]
hUC-MSC	2.5 × 10^12^ particles/dose	single	Portal vein	Liver IRI mouse model	Yes	Inhibit apoptosis and inflammation (the inflammatory factors TNF-α, IL-6a, and IL-1β are significantly reduced)	Transfer miR-1246 targeting the *GSK3β*/Wnt/β-catenin pathway	Xie et al., 2019 [15]
hUC-MSC	Not provided	single	Not provided	Liver IRI rat model	Yes	Inhibit autophagy and apoptosis	By secreting miR-20a, it targets *Fas* and *Beclin-1* and inhibits their expression	Zhang et al., 2020 [31]
hUC-MSC	0.4 μg/dose	single	Caudal vein	Partial hepatectomy rat model	Yes	Promote hepatocyte proliferation (the proliferation marker PCNA was elevated)	The expression of *Foxg1* is downregulated by the secretion of miR-124	Song et al., 2021 [32]
hUC-MSC	3 mg/dose	single	Caudal vein	Liver IRI rat model	Yes	Inhibit oxidative stress (increased levels of the mitochondrial antioxidant enzyme MnSOD); inhibit inflammation (prevent neutrophils from entering the inflammatory microenvironment)	Not assessed	Yao et al., 2019 [33]
hUC-MSC	100 μg/dose	single	Caudal vein	Liver IRI mouse model	No	Reduce autophagy and apoptosis	Transfer of mitochondria to modulate the formation of NETs	Lu et al., 2022 [34]
hUC-MSC	8, 16, and 32 mg/kg	single	Caudal vein/gavage	CCl_4_-induced liver injury mouse model	Yes	Inhibit oxidative stress; reduce apoptosis	Phosphorylation of ERK1/2 is induced by the secretion of GPX1	Yan et al., 2017 [35]
mBM-MSC	2 × 10^10^ particles/dose	single	Caudal vein	Liver IRI mouse model	Yes	Inhibit inflammation (decreased expression of inflammatory cytokines IL-6 and IL-1β); reduce apoptosis (reduction in caspase-3 positive cells and apoptotic cells)	Targeting Nlrp12	Haga et al., 2017 [36]
hBM-MSC	1 × 10^9^ particles/dose	single	Vena cava inferior	Liver IRI mouse model	No	Reduce inflammation; reduce liver damage	Not assessed	Anger et al., 2019 [37]
mBM-MSC	Not provided	once daily for 3 days	Caudal vein	Liver IRI mouse model	Yes	Ba-EVs can improve Th17/Treg imbalance	Inducing *FGF21* expression, inhibiting the *JAK2/STAT3* pathway, and activating *FOXO1* expression	Zhang et al., 2024 [38]
mBM-MSC	50 μg/dose	single	Caudal vein	Liver IRI mouse model	Yes	Inhibit cell apoptosis	Regulates the p53 signaling pathway through *PTEN*	Li et al., 2023 [39]
mBM-MSC-Heps	100 μg/dose	before and after the operation	Caudal vein	Liver IRI mouse model	Yes	Reduce apoptosis; reduce liver damage	Enhanced autophagy	Yang et al., 2020 [40]
mBM-MSC	20 μg/dose	single	Arteria femoralis	Traumatic hemorrhagic shock (THS)-induced liver injury mouse model	Yes	Inhibit inflammation	By delivering IL-10 and interacting with Kupffer cells, it causes Kupffer cells to change to an anti-inflammatory phenotype (M2)	Zhang et al., 2022 [41]
BM-MSC	150 µg/dose	single	Caudal vein	D-GaIN/LPS induced ALI mouse model	Yes	Inhibit ROS production and lipid peroxide-induced iron death	Ba-EVs inhibit iron death by activating the Keap1-NRF2 pathway through P62	Zhao et al., 2022 [42]
hBM-MSC	Not provided	single	Intraperitoneal injection	Liver IRI rat model	Yes	Inhibit inflammation and apoptosis	Not assessed	Wei et al., 2020 [43]
mBM-MSC	10 μg/dose	0, 8, and 16 h after con-A injection	Vein	Concanavalin A-induced liver injury mouse model	Yes	Inhibit inflammation; reduce apoptosis	Not assessed	Tamura et al., 2016 [23]
rBM-MSC	50 μg/dose	single	Portal vein	Liver IRI rat model; CCl_4_-induced ALI rat model	Yes	Reduce oxidative stress and apoptosis	Not assessed	Damania et al., 2018 [44]
Mini-pig AD-MSC	100 µg/dose	single	Vein	Liver IRI rat model	Yes	Inhibit inflammation (the inflammation markers MIF, MMP-9, L-1β, TNF-α, and COX-2 are decreased); inhibit oxidative stress (NOX-1 and NOX-2 levels decreased; HO-1 and NQO-1 levels increased); inhibit apoptosis (reduce caspase-3 and PARP)	Not assessed	Sun et al., 2017 [45]
rAD-MSC	30 μg/dose	single	Portal vein	Liver IRI rat model	Yes	Reduce apoptosis and oxidative stress; reduce inflammation	Phosphorylation of ERK1/2 and GSK-3β by prostaglandin E2 (PGE2)	Zhang et al., 2022 [46]
rAD-MSC	100 µg/dose	single	Caudal vein	Liver IRI rat model	No	Inhibit oxidative stress (MDA, ROS oxidation index decreased, content of antioxidant enzymes SOD, CAT, and GSH-px increased), reduce cell apoptosis (inhibition of caspase-3 and caspase-9 activities, decrease Bax mRNA and protein expression, increase Bcl-2 mRNA and protein expression)	Reduce mitochondrial division, promote mitochondrial fusion, and improve mitochondrial biosynthesis	Zhang et al., 2021 [1]
hAD-MSC	250 µg/dose	single	Caudal vein	CCl_4_-induced liver injury mouse model	Yes	Promote hepatocyte proliferation	Not assessed	Gupta et al., 2022 [47]
mAD-MSC	200 μL	single	Caudal vein	CCl_4_-induced ALI mouse model	Yes	EVs loaded with vitamin A and quercetin were more effective in reducing liver damage	Not assessed	Fang and Liang, 2021 [48]
hAD-MSC	Not provide	single	Caudal vein	Liver IRI rat model	Yes	Reduce liver injury	Through the miR-183/*ALOX5* axis	Gong et al., 2023 [49]
hAD-MSC	100 µg/dose	single	Caudal vein	Liver IRI rat model	No	Inhibit cell pyroptosis; promote hepatocyte proliferation	Inhibit the NF-κB pathway and activate the Wnt/β-catenin pathway	Piao et al., 2022 [50]
mini-pig AD-MSC	5 × 10^9^ particles/kg	single	Portal vein	Liver IRI mini-pig model	Yes	Inhibit apoptosis, pyroptosis, and inflammatory responses	Not assessed	Wang et al., 2023 [51]
rAD-MSC	100 µg/dose	single	Caudal vein	Liver IRI rat model	No	Inhibit ERS and inflammation	Not assessed	Zhang et al., 2023 [52]
mini-pig AD-MSC	5 × 10^9^ particles/kg	single	Portal vein	Liver IRI mini-pig model	Yes	Inhibit inflammation; promote hepatocyte proliferation	Not assessed	Wang et al., 2024 [53]
mini-pig AD-MSC	5 × 10^9^ particles/kg	single	Portal vein	Liver IRI mini-pig model	No	Modulate the ERS response	Not assessed	Wang et al., 2023 [54]
hiPSC-MSC	600 µg/dose	single	Vena cava inferior	Liver IRI rat model	No	Inhibit inflammation (TNF-α, IL-6, and HMGB1 decreased significantly), reduce oxidative stress (increased GSH, GSH-px, and SOD levels), reduce apoptosis (significantly decreased caspase-3 and bax levels), and promote hepatocyte proliferation	Not assessed	Nong et al., 2016 [55]
hiPSC-MSC	2.5 × 10^12^ particles/dose	single	Vena cava inferior	Liver IRI mouse model	Yes	Reduce liver damage (significantly decreased AST and ALT levels) and promote hepatocyte proliferation (significantly increased expression of the proliferation markers PCNA and PHH3)	The sphingosine kinase and sphingosine-1-phosphate-dependent pathway	Du et al., 2017 [56]
HLSC	5 or 25 × 10^8^ particles/g liver tissue	single	The NMP circuit	Liver long warm ischemia rat model	Yes	Promote hepatocyte regeneration; damage mitigation	Not assessed	De Stefano et al., 2021 [57]
HLSC	5 × 10^8^ particles/g liver tissue	single	The NMP circuit	Liver short warm ischemia rat model	No	Reduce liver damage	Not assessed	Rigo et al., 2018 [58]
HLSC	3 or 7.5 × 10^9^ particles/dose	single	Vein	Liver IRI mouse model	No	Reduce liver damage; reduce inflammation	Not assessed	Calleri et al., 2021 [59]

Note: ES-MSC: embryonic stem cell–mesenchymal stem cell; PCNA: proliferating cell nuclear antigen; 3D-hESC: 3D human embryonic stem cell; TGFβRII: Transforming Growth Factor-β type II receptor; HSC: hepatic stellate cell; hUC-MSC: human umbilical cord-derived mesenchymal stem cell; NFAT1: nuclear factor of activated T cells 1; STAT3: signal transducer and activator of transcription 3; Th17: T helper 17 cell; Treg: regulatory T cell; *GSK3β*: glycogen synthase kinase 3β; TNF-α: tumor necrosis factor alpha; IL-6: interleukin 6; MnSOD: manganese superoxide dismutase; NETs: neutrophil extracellular traps; GPX1: glutathione peroxidase 1; ERK1/2: Recombinant Extracellular Signal-Regulated Kinase 1/2; Nlrp12: NACHT, LRR, and PYD domain-containing protein 12; mBM-MSC: mouse bone marrow-derived mesenchymal stem cell; mBM-MSC-Heps: mouse bone marrow–mesenchymal stem cell-derived hepatocyte-like cells; Ba-EVs: extracellular vesicles derived from baicalin-pretreated MSCs; ROS: reactive oxygen species; hBM-MSC: human bone marrow–mesenchymal stem cell; AD-MSC: adipose mesenchymal stem cell; rAD-MSC: rat adipose mesenchymal stem cell; hAD-MSC: human adipose mesenchymal stem cell; mAD-MSC: mouse adipose mesenchymal stem cell; mini-pig AD-MSC: mini-pig adipose mesenchymal stem cell; ERS: endoplasmic reticulum stress; MIF: migration inhibitory factor; MMP-9: matrix metallopeptidase 9; IL-1β: interleukin-1 beta; COX-2: cyclooxygenase-2; NOX-1: NADPH oxidase 1; NOX-2: NADPH oxidase 2; PARP: Poly ADP ribose polymerase; PGE2: prostaglandin E2; GSK-3β: glycogen synthase kinase-3b; MDA: malondialdehyde; SOD: superoxide dismutase; CAT: catalase; GSH-px: glutathione peroxidase; hiPSC-MSC: human induced pluripotent stem cell-derived mesenchymal stromal cell; HMGB1: high-mobility group box 1; AST: aspartate aminotransferase; ALT: alanine aminotransferase; PHH3: phosphohistone-H3; HLSC: human liver stem cell; NMP: normothermic machine perfusion.

**Table 2:** Clinical research progress in the use of EVs derived from stem cells to treat liver injury.

Registration Number	Title	Country	Year	Status	Study Type	Phase	EV Source
NCT05940610	The Safety and Efficacy of MSC-EVs in Acute/Acute-on-Chronic Liver Failure	China	2023	Withdrawn	Interventional	1, 2	hMSCs
NCT05881668	MSC-EV in Acute-on-Chronic Liver Failure After Liver Transplantation	China	2023	Withdrawn	Interventional	1	MSCs
NCT05871463	Effect of Mesenchymal Stem Cells-derived Exosomes in Decompensated Liver Cirrhosis	Iran	2023	Recruiting	Interventional	2	hUC-MSCs
ChiCTR-INR-17010677	Study on the effect of MSCs-HNF4α exosomes combined with normal mechanical perfusion on liver transplantation of fatty liver	China	2017	Not yet recruiting	Interventional	New Treatment Measure Clinical Study	hMSCs-HNF4α
ChiCTR2300075676	A small sample clinical study of the safety and initial efficacy of exosomes in the treatment of cirrhosis	China	2023	Recruiting	Interventional	New Treatment Measure Clinical Study	MB-MSCs
ChiCTR1800020076	A clinical study for cancer stem cells exosome loaded dendritic cells vaccine and its activated CTL injection in the treatment of hepatic cell cancer and other solid tumors	China	2018	Not yet recruiting	Interventional	1, 2	cancer stem cells

Note: hMSCs-HNF4α: human mesenchymal stem cells–Hepatocyte Nuclear Factor 4 Alpha; MB-MSC: menstrual blood mesenchymal stem cell.

## Data Availability

No new data were created or analyzed in this study. Data sharing is not applicable to this article.
